# Characteristics of Tumor-Infiltrating Lymphocytes Prior to and During Immune Checkpoint Inhibitor Therapy

**DOI:** 10.3389/fimmu.2020.00364

**Published:** 2020-03-04

**Authors:** Ioana Plesca, Antje Tunger, Luise Müller, Rebekka Wehner, Xixi Lai, Marc-Oliver Grimm, Sergio Rutella, Michael Bachmann, Marc Schmitz

**Affiliations:** ^1^Faculty of Medicine Carl Gustav Carus, Institute of Immunology, TU Dresden, Dresden, Germany; ^2^National Center for Tumor Diseases (NCT), Partner Site Dresden, Dresden, Germany; ^3^German Cancer Consortium (DKTK), Partner Site Dresden, German Cancer Research Center (DKFZ), Heidelberg, Germany; ^4^Department of Urology, Jena University Hospital, Jena, Germany; ^5^John van Geest Cancer Research Center, College of Science and Technology, Nottingham Trent University, Nottingham, United Kingdom; ^6^Department of Radioimmunology, Institute of Radiopharmaceutical Cancer Research, Helmholtz Center Dresden-Rossendorf, Dresden, Germany

**Keywords:** cancer immunotherapy, immune architecture, immune monitoring, immune checkpoint inhibition, cytotoxic T lymphocyte antigen 4, programmed cell death protein 1, programmed cell death 1 ligand 1

## Abstract

The tumor immune contexture plays a major role for the clinical outcome of patients. High densities of CD45RO^+^ T helper 1 cells and CD8^+^ T cells are associated with improved survival of patients with various cancer entities. In contrast, a higher frequency of tumor-infiltrating M2 macrophages is correlated with poor prognosis. Recent studies provide evidence that the tumor immune architecture also essentially contributes to the clinical efficacy of immune checkpoint inhibitor (CPI) therapy in patients. Pretreatment melanoma samples from patients who experienced a clinical response to anti-programmed cell death protein 1 (PD-1) treatment show higher densities of infiltrating CD8^+^ T cells compared to samples from patients that progressed during therapy. Anti-PD-1 therapy results in an increased density of tumor-infiltrating T lymphocytes in treatment responders. In addition, elevated frequencies of melanoma-infiltrating TCF7^+^CD8^+^ T cells are correlated with beneficial clinical outcome of anti-PD-1-treated patients. In contrast, a high density of tumor-infiltrating, dysfunctional PD-1^+^CD38^hi^ CD8^+^ cells in melanoma patients is associated with anti-PD-1 resistance. Such findings indicate that comprehensive tumor immune contexture profiling prior to and during CPI therapy may lead to the identification of underlying mechanisms for treatment response or resistance, and the design of improved immunotherapeutic strategies. Here, we focus on studies exploring the impact of intratumoral T and B cells at baseline on the clinical outcome of CPI-treated cancer patients. In addition, recent findings demonstrating the influence of CPIs on tumor-infiltrating lymphocytes are summarized.

## Introduction

Accumulating evidence indicates that the tumor immune contexture plays a critical role for the clinical outcome of cancer patients (1–4). Major components of the tumor immune architecture are CD8^+^ and CD4^+^ T cells that can essentially contribute to tumor elimination. Activated CD8^+^ T cells produce large amounts of proinflammatory cytokines such as tumor necrosis factor (TNF)-α and interferon (IFN)-γ and exhibit a profound tumor-directed cytotoxicity. Stimulated CD4^+^ T cells secrete various cytokines that promote the differentiation of B cells into antibody-producing plasma cells (5). They also enhance the capacity of dendritic cells (DCs) to induce CD8^+^ T cell responses and can eliminate tumor cells directly (5). When analyzing the clinical relevance of tumor-infiltrating T cells, it has been demonstrated that high densities of CD4^+^ memory T helper (T_H_) 1 cells and CD8^+^ T cells are associated with improved disease-free and overall survival (OS) of colorectal cancer patients (6, 7). Recently, a multi-center study has been initiated to assess the prognostic value of tumor-infiltrating T cell numbers in colon cancer patients (8). Patients with a so-called high Immunoscore, which is characterized by a high frequency of CD3^+^ and CD8^+^ T cells in the tumor center and the invasive margin, had the longest survival and the lowest risk of recurrence (8). These results suggest that the Immunoscore may represent a reliable estimate of the risk of disease recurrence and support its implementation in the classification of colon cancer. In addition to colorectal cancer patients, a correlation between high densities of T_H_1 cells or CD8^+^ T cells and good prognosis has also been reported for patients with other cancer entities (1, 3).

Macrophages and DCs are other key components of the tumor immune contexture that can profoundly influence tumor growth and spreading. Macrophages can be classified according to their phenotype and functional properties (9, 10). M1 macrophages, which express high levels of proinflammatory mediators such as TNF-α, interleukin (IL)-1β, reactive oxygen species, and nitric oxide, act in a tumoricidal manner. Based on their tumor-directed properties, M1 macrophages are generally associated with a favorable clinical outcome of cancer patients (1, 3). In contrast, M2 macrophages, which are characterized by the release of pro-angiogenic mediators such as vascular endothelial growth factor (VEGF) and immunosuppressive cytokines such as IL-10 and transforming growth factor-β, are generally correlated with poor prognosis among cancer patients (1, 3). DCs display an extraordinary capacity to induce and regulate T cell responses and efficiently enhance the immunomodulatory and cytotoxic potential of natural killer (NK) cells (11). Due to these functional capabilities, DCs play a major role in antitumor immunity. When investigating the clinical impact of blood DC subsets, it has been demonstrated that a higher expression of specific gene signatures for myeloid DC1 and DC2 as well as for plasmacytoid DCs are associated with a higher probability for disease-free survival of patients with luminal breast cancer (12). Furthermore, a higher DC1-specific gene signature was significantly associated with improved survival in patients with various cancer entities (13). However, tumor-infiltrating DCs can also be defective in their functional activity and can contribute to immune suppression (14). For example, we have shown that a higher density of 6-sulfo LacNAc monocytes (slanMo), representing a subset of human non-classical blood monocytes that can differentiate into DCs (15), is significantly associated with a poor prognosis of clear cell renal cell cancer (RCC) patients (16). The tumor-infiltrating slanMo displayed an immature phenotype and expressed IL-10, which may explain this correlation.

Recent studies revealed that the tumor immune contexture also essentially contributes to the clinical efficacy of immune checkpoint inhibitor (CPI) therapy that evolved as a very promising treatment modality for cancer patients (17). Antibody-mediated blockade of the immune checkpoint receptors cytotoxic T lymphocyte antigen 4 (CTLA-4), programmed cell death protein 1 (PD-1) or programmed cell death 1 ligand 1 (PD-L1) resulted in objective clinical responses and enhanced survival of cancer patients (18–20). Here, the current knowledge about the impact of intratumoral T and B cells at baseline on the clinical outcome of CPI-treated patients and treatment-mediated effects on tumor-infiltrating lymphocytes is summarized.

## Characteristics of Intratumoral T Cells Prior to and During Anti-CTLA-4 Therapy

### Function and Therapeutic Targeting of CTLA-4

Cytotoxic T lymphocyte antigen 4 is a member of the immunoglobulin superfamily, which is induced on the surface of T cells by antigen binding to the T cell receptor (21–23). CTLA-4 competes with CD28 for binding to CD80 or CD86 on professional antigen-presenting cells (APCs). Thereby it binds CD80 and CD86 more tightly than CD28 and delivers a negative signal, which dampens the early T cell activation. CTLA-4 regulates the amplitude of CD4^+^ T cell priming and also the CD4^+^ T cell help for the induction of CD8^+^ T cell responses in lymphoid tissues. CTLA-4 is constitutively expressed on regulatory T (T_reg_) cells, enhancing their immunosuppressive activity (24). Accordingly, CTLA-4 blockade fosters the expansion, cytokine secretion, and cytotoxic potential of T effector cells and inhibits the immunosuppressive activity of T_reg_ cells, resulting in improved antitumor responses. Therefore, CTLA-4 blockade is an attractive immunotherapeutic strategy to significantly enhance effector T cell-mediated antitumor immunity (25). Two phase III clinical trials have been conducted to explore the therapeutic efficacy of the anti-CTLA-4 monoclonal antibody ipilimumab. Melanoma patients treated with ipilimumab with or without a glycoprotein 100 peptide vaccine showed significantly improved OS compared to patients receiving the peptide vaccine alone (26). Furthermore, the combination of the DNA-alkylating agent dacarbazin with ipilimumab led to improved OS in melanoma patients compared to dacarbazin alone (27). Based on these clinical trials, ipilimumab was approved by the United States Food and Drug Administration (FDA) for the treatment of patients with metastatic melanoma in 2011 (28).

### Correlation Between Frequency and Phenotype of Intratumoral T Cells and Clinical Efficacy of CTLA-4 Blockade

Recently, the association between immunological parameters in tumor tissues at baseline and the clinical activity of anti-CTLA-4 therapy has been explored. Surprisingly, Hamid et al. found a positive correlation between clinical efficacy of CTLA-4 blockade and a high baseline expression of either the T_reg_ cell-associated transcription factor FoxP3 or the immunosuppressive molecule indoleamine 2,3-dioxygenase (IDO) in melanoma patients (29). Whereas no correlation between the frequency of pre-existing tumor-infiltrating T cells and clinical activity was observed, an anti-CTLA-4 therapy-mediated increase of the intratumoral T cell density was associated with improved clinical outcome. Various studies further substantiate the influence of anti-CTLA-4 treatment on the frequency and phenotype of intratumoral T cells. Thus, CTLA-4 blockade resulted in a significant increase of CD8^+^ T cells regardless of clinical responses in melanoma patients (30). Hodi et al. observed clinical responses in the majority of metastatic melanoma patients who received ipilimumab after vaccination with irradiated, autologous tumor cells engineered to secrete granulocyte-macrophage colony-stimulating factor (GM-CSF) (31). Analysis of posttreatment biopsies from metastatic lesions revealed a relation between the extent of therapy-induced tumor necrosis and the natural logarithm of the ratio of tumor-infiltrating CD8^+^ effector T cells to T_reg_ cells, suggesting that ipilimumab can alter the balance of effector T cells and T_reg_ cells (31). When investigating anti-CTLA-4 therapy-related effects on the density of tumor-infiltrating T_reg_ cells, Sharma et al. found that this treatment does not significantly modulate the frequency of T_reg_ cells in patients (32).

**FIGURE 1:**
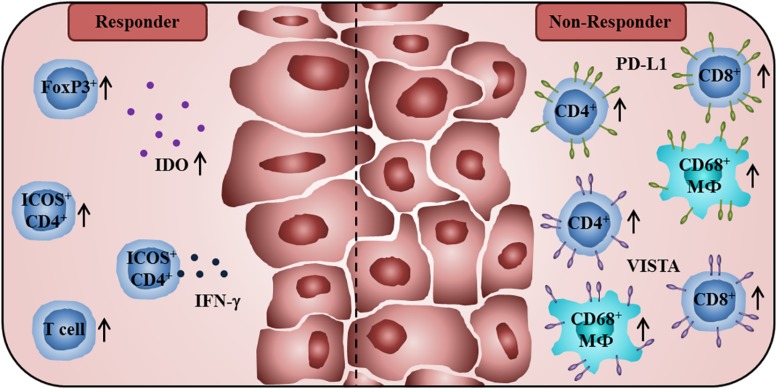
Immunological characteristics of tumor patients receiving anti-CTLA-4 antibodies associated with improved clinical outcome or therapy resistance. A high baseline expression of T_reg_ cell-associated FoxP3 and IDO, and a treatment-induced increase of tumor-infiltrating T lymphocytes are associated with better clinical efficacy of CTLA-4 blockade. Anti-CTLA-4 therapy enhances the frequency of intratumoral ICOS^+^CD4^+^ T cells that is correlated with better OS. A proportion of these ICOS^+^CD4^+^ T cells is characterized by the production of IFN-γ. Non-responders to anti-CTLA-4 therapy show a higher percentage of PD-L1- or VISTA-expressing CD4^+^ T cells, CD8^+^ T lymphocytes, and CD68^+^ macrophages in posttreatment tumor samples.

In further studies, the impact of anti-CTLA-4 therapy on the phenotype of intratumoral T cells has been explored. It has been reported that this therapeutic strategy enhances the density of tumor-infiltrating CD4^+^ T cells expressing the costimulatory molecule inducible T cell costimulator (ICOS) (33). In addition, a subset of IFN-γ-producing T cells was detected within the ICOS^+^CD4^+^ T cell population, indicating that anti-CTLA4 therapy can induce a T_H_1 polarization in CD4^+^ effector cells (33). Wei et al. observed an expansion of tumor-infiltrating ICOS^+^ T_H_1-like CD4^+^ T cells and exhausted-like CD8^+^ T cells following anti-CTLA-4 blockade in melanoma patients (34). Moreover, an enhanced frequency of melanoma-infiltrating ICOS^+^ CD4^+^ T cells, sustained over 3 months of anti-CTLA-4 treatment, was associated with better OS (35). When evaluating tissue specimens from prostate cancer patients prior to and after anti-CTLA-4 blockade, Gao et al. detected a higher proportion of tumor-infiltrating CD4^+^ T cells, CD8^+^ T lymphocytes, and CD68^+^ macrophages expressing PD-L1 or V-domain Ig suppressor of T cell activation (VISTA), representing another inhibitory immune checkpoint receptor (36), after treatment (37). PD-L1 and VISTA expression on these immune cell subsets may contribute to the poor responsiveness of prostate cancer patients to anti-CTLA-4 therapy. A summary of immune cell characteristics that may have an impact on the clinical efficacy of anti-CTLA-4 therapy is given in Figure 1.

## Characteristics of Tumor-Infiltrating Lymphocytes Prior to and During Anti-PD-1/PD-L1 Treatment

### Function and Therapeutic Targeting of the PD-1/PD-L1 Axis

Programmed cell death protein 1 is another immune checkpoint receptor of the immunoglobulin superfamily, which can be found on activated T effector cells, NK cells, and B cells (18, 38). PD-1 is also expressed by T_reg_ cells and fosters their proliferation after ligand binding (39). PD-L1 and PD-L2 represent the ligands for PD-1, the latter having a higher affinity to PD-1. PD-L1 can be widely detected on tumor cells as well as hematopoietic and non-hematopoietic cells and its expression is inducible by proinflammatory cytokines such as IFN-γ. PD-L2 is characterized by a more restricted expression pattern, being mainly detectable on APCs and induced mostly by IL-4 and GM-CSF (40–43). Besides PD-1, PD-L1 can also bind to CD80 on T cells, thereby delivering another inhibitory signal (44). The main role of PD-1 is to modulate important functional properties of antigen-experienced effector T cells within the peripheral tissues. Thus, expansion, cytokine release, and cytotoxic activity of stimulated T cells are inhibited upon interaction of PD-1 with its ligands, protecting the tissue from collateral damage during immune response (40, 45–47). This pathway is adopted by tumors leading to prevention from immune attack. Therefore, anti-PD-1 and anti-PD-L1 antibodies have been developed to enhance T cell-mediated antitumor immunity. The application of such antibodies induced objective clinical responses and improved survival in cancer patients (48–50). Consequently, the FDA approved anti-PD-1/PD-L1 therapy for various tumor entities (28, 51).

### Correlation Between PD-L1 Expression by Tumor Cells and Tumor-Infiltrating Immune Cells and Clinical Efficacy of PD-1/PD-L1 Blockade

Various clinical trials clearly indicated that PD-L1 expression by tumor-infiltrating immune cells and tumor cells significantly influences the efficacy of anti-PD-1/PD-L1 treatment. Accordingly, an association between intratumoral PD-L1 expression in pretreatment tissue specimens and objective clinical responses in anti-PD-1/PD-L1-treated cancer patients has been reported (52). Herbst et al. demonstrated that a high level of intratumoral PD-L1, particularly when detected on tumor-infiltrating immune cells, was associated with clinical responses in anti-PD-L1 antibody-treated cancer patients (53). Topalian et al. observed that 9 of 25 patients with PD-L1^+^ tumors experienced an objective clinical response, whereas none out of 17 patients with PD-L1^–^ tumors achieved an objective response (54). In agreement with these findings, it has been reported that PD-L1 expression in at least 50% of tumor cells correlates with improved efficacy of anti-PD-1 therapy in non-small-cell lung cancer (NSCLC) patients (49). Further clinical trials yielded contradictory results (52). Motzer et al. investigated a large cohort of RCC patients undergoing anti-PD-1 therapy and found a reduced OS for patients with 1% or greater intratumoral PD-L1 expression compared to patients with less than 1% (50). Gettinger et al. did also not find a clear correlation between PD-L1 expression and clinical response or survival in anti-PD-1-treated NSCLC patients (55). However, the results are not always comparable since various assays, antibodies, cut-off values, and different scoring methods are utilized to determine PD-L1^+^ cells by immunohistochemistry.

### Association Between Frequency and Phenotype of Tumor-Infiltrating T Cells and Clinical Efficacy of PD-1/PD-L1 Blockade

Recent studies revealed that the density and phenotype of tumor-infiltrating T cells play an essential role for the clinical efficacy of anti-PD-1/PD-L1 therapy. Using melanoma tissue samples collected before and during treatment with anti-PD-1 antibodies, Tumeh et al. determined the frequency of tumor-infiltrating CD8^+^ T cells (56). A higher density of melanoma-infiltrating CD8^+^ T cells at baseline was indicative of responding patients, suggesting that pre-existing intratumoral CD8^+^ T cells are predictors of a clinical response to anti-PD-1 therapy. This finding was further substantiated by another study, investigating RCC tissues from patients treated with anti-PD-L1 and anti-VEGF antibodies (57). McDermott et al. found a correlation between a high T effector gene signature expression at baseline and an improved overall response rate and progression-free survival (PFS) of the treated patients. In contrast, a high myeloid inflammation gene signature expression was associated with reduced PFS in patients receiving anti-PD-L1 alone or anti-PD-L1 and anti-VEGF antibodies. When performing an in-depth analysis of intratumoral CD8^+^ T lymphocytes in NSCLC patients, Thommen et al. described three distinct CD8^+^ T cell subsets based on PD-1 expression (58). In addition to CD8^+^ T cell subpopulations with intermediate (PD-1^N^) and no PD-1 expression, a subset with high PD-1 expression (PD-1^T^) was identified that displayed a markedly different transcriptional and metabolic profile. The PD-1^T^ CD8^+^ T cells are characterized by the secretion of CXCL13 that can mediate recruitment of follicular T_H_ cells and B cells to the tumor microenvironment and may also foster the formation of intratumoral tertiary lymphoid structures (TLS). The presence of PD-1^T^ T cells emerged as a strong predictor for the clinical outcome of anti-PD-1-treated NSCLC patients (58).

The impact of anti-PD-1 therapy on the phenotype and frequency of intratumoral T cells was also explored. Melanoma patients who responded to anti-PD-1 therapy showed an increased intratumoral CD8^+^ T cell density that was associated with radiographic reduction of tumor size (56). In another study, two major intratumoral CD8^+^ T cell states that were associated with clinical response have been identified in melanoma patients treated with PD-1- and/or CTLA-4 blockade (59). Single-cell RNA sequencing resulted in the identification of intratumoral CD8^+^ T cells with increased expression of genes linked to memory, activation, and cell survival that were enriched in responding melanoma lesions. In contrast, CD8^+^ T cells with increased expression of genes linked to exhaustion were enriched in non-responding lesions. Thus, the ratio of memory-like to exhausted CD8^+^ T cells was linked with clinical outcome. In addition, elevated levels of melanoma-infiltrating TCF7^+^CD8^+^ T cells predicted clinical benefit in anti-PD-1-treated patients (59). By using a tumor mouse model, Siddiqui et al. showed that intratumoral TCF7^+^PD-1^+^CD8^+^ T cells with stem-like properties can mediate tumor control to CPI therapy (60). In addition, melanoma patients treated with anti-CTLA-4 and/or anti-PD-1-antibodies showed a higher proportion of intratumoral TCF7^+^PD-1^+^CD8^+^ T cells than untreated patients (60). Furthermore, an increased density of TCF7^+^PD-1^+^CD8^+^ T cells at baseline was associated with prolonged survival in melanoma patients treated with anti-CTLA-4 and anti-PD-1-antibodies (61). Moreover, Verma et al. reported that the status of CD8^+^ T cell priming essentially influences anti-PD-1 therapeutic resistance (62). Thus, administration of anti-PD-1 antibodies in unprimed or suboptimal primed CD8^+^ T cell conditions led to the generation of dysfunctional PD-1^+^CD38^hi^CD8^+^ cells that contribute to PD-1 blockade resistance and treatment failure. However, the induction of dysfunctional CD8^+^ cells was prevented and treatment resistance was reversed when anti-PD-1 therapy was applied to optimally primed CD8^+^ T lymphocytes. They also found that a high density of tumor-infiltrating PD-1^+^CD38^hi^CD8^+^ cells in melanoma patients can serve as a biomarker of anti-PD-1 resistance. Zappasodi et al. described an intratumoral accumulation of CD4^+^FoxP3^–^PD-1^hi^ T cells (4PD-1^hi^) in immunotherapy-naïve melanoma and NSCLC patients (63). These T cells were shown to inhibit the proliferation and activation of T effector cells. In addition, the authors found that a lack of effective 4PD-1^hi^ reduction after PD-1 blockade correlates with poor prognosis (63).

### Impact of the Frequency of Tumor-Infiltrating B Cells and TLS on Clinical Efficacy of Anti-PD-1 Therapy

**FIGURE 2:**
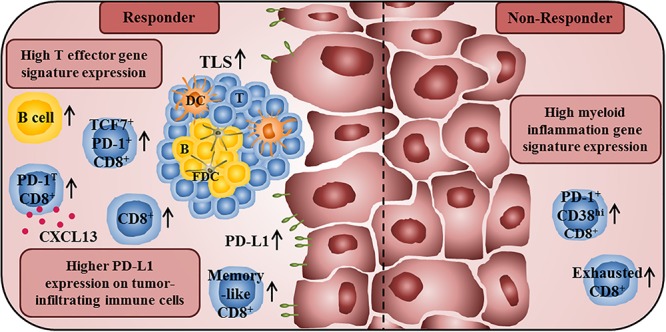
Immune profile of anti-PD-1/PD-L1 antibody-treated tumor patients associated with improved clinical outcome or therapy resistance. A high T effector gene signature expression in pretherapy tumor samples is associated with improved survival of anti-PD-L1- and anti-VEGF-treated cancer patients. In addition, responders to anti-PD-1 treatment show a higher frequency of intratumoral CD8^+^ T cells at baseline and an increased frequency of tumor-infiltrating CD8^+^ T cells during therapy. Furthermore, they also have a higher proportion of intratumoral memory-like CD8^+^ T cells. The presence of PD-1^T^ CD8^+^ T cells, which are characterized by a high PD-1 expression and by the capability to secrete CXCL13, is also correlated with improved clinical outcome of anti-PD-1-treated cancer patients. Moreover, an increased frequency of TCF7^+^PD-1^+^CD8^+^ T cells in pretreatment tumor samples is associated with prolonged survival in patients treated with anti-CTLA-4 and anti-PD-1-antibodies. An increased density of B cells and TLS, consisting of a DC-containing T cell zone and a follicular DC-containing B cell zone, in pretreatment tumor samples is also correlated with an increased survival of anti-PD-1-treated patients. Furthermore, a higher PD-L1 expression on tumor cells and tumor-infiltrating immune cells is correlated with better clinical responses to anti-PD-1/PD-L1 therapy. In contrast, high frequencies of exhausted CD8^+^ T cells and PD-1^+^CD38^hi^CD8^+^ T cells in tumor tissues are associated with resistance to anti-PD-1 therapy. Non-responders to anti-PD-L1 and anti-VEGF therapy also show a high myeloid inflammation gene expression signature.

Emerging evidence suggests that tumor-infiltrating B cells play an important role for the clinical outcome of anti-PD-1-treated cancer patients. Thus, a higher frequency of melanoma-infiltrating B cells with a plasmablast-like phenotype before therapy was associated with improved patient survival to anti-PD-1 treatment (64). More recently, Petitprez et al. observed that the sarcoma immune class E, which is characterized by TLS containing T cells, follicular DCs, and a high density of B cells, is correlated with an improved response rate and survival to PD-1 blockade (65). In addition, a higher density of tumor-infiltrating B cells and TLS has been detected in treatment responders in a cohort of melanoma patients receiving anti-PD-1-antibodies alone or combined with anti-CTLA-4 antibodies in a neoadjuvant setting (66). The importance of tumor-associated TLS for the clinical efficacy of anti-PD-1 treatment is further supported by another clinical trial, demonstrating that a higher TLS density at baseline was correlated with increased survival of melanoma patients (67). An overview about immune cell characteristics that may influence the clinical efficacy of anti-PD-1/PD-L1 therapy is given in Figure 2.

## Conclusion

The location, density, and functional orientation of tumor-infiltrating immune cells play a critical role for the clinical outcome of cancer patients. Thus, high frequencies of CD4^+^ T_H_1 cells and CD8^+^ T cells in the tumor center and the invasive margin were associated with improved OS of colorectal cancer patients. Whereas M1 macrophages were correlated with a favorable clinical outcome of cancer patients with various cancer entities, M2 macrophages were generally associated with poor prognosis. Such findings indicate that tumor-infiltrating immune cells can significantly influence tumor growth and spreading. Recent studies revealed that the tumor immune contexture also essentially contributes to the clinical efficacy of CTLA-4 or PD-1/PD-L1 blockade that induced objective clinical responses and improved survival in patients with various tumor types. However, a significant number of patients do not respond to CPI therapy. Therefore, deciphering the immunogenicity of the tumor cells and the tumor immune architecture prior to and during CPI therapy may lead to the discovery of novel modes of action or resistance and to the design of improved treatment modalities for cancer patients. For example, it has been demonstrated that a limited presentation of tumor-associated neoepitopes by tumor cells and the lack of pre-existing intratumoral T cells are associated with poor responsiveness of cancer patients to CPI therapy. Therefore, other treatment modalities that increase the expression of components of the antigen-processing and presentation machinery and the neoantigen load of tumor cells as well as promote T cell trafficking to tumor tissues are required to improve the clinical response rate to current CPI therapy. Promising treatment options comprise radiotherapy as well as the application of chemotherapeutic agents and epigenetic drugs that can efficiently increase tumor cell immunogenicity and stimulate antitumor immune responses. Vaccination strategies including neoantigens and the administration of non-modified or engineered T cells can increase the frequency of tumor-infiltrating and -reactive T lymphocytes. The intratumoral application of oncolytic viruses or adjuvants can also improve CPI-based therapies by direct tumor cell elimination, the recruitment of DCs and T cells to the tumor, and the activation of innate and adaptive antitumor immunity.

## Author Contributions

IP and AT drafted the manuscript. LM, RW, XL, M-OG, SR, MB, and MS reviewed and edited the manuscript.

## Conflict of Interest

The authors declare that the research was conducted in the absence of any commercial or financial relationships that could be construed as a potential conflict of interest.
